# Effects of Optically Imposed Astigmatism on Early Eye Growth in Chicks

**DOI:** 10.1371/journal.pone.0117729

**Published:** 2015-02-12

**Authors:** Chin Hung Geoffrey Chu, Chea Su Kee

**Affiliations:** School of Optometry, The Hong Kong Polytechnic University, Hung Hom, Kowloon, Hong Kong SAR, China; National Eye Institute, UNITED STATES

## Abstract

**Purpose:**

To determine the effects of optically imposed astigmatism on early eye growth in chicks.

**Methods:**

5-day-old (P5) White Leghorn chicks were randomly assigned to either wear, monocularly, a “high magnitude” (H: +4.00DS/-8.00DC) crossed-cylindrical lens oriented at one of four axes (45, 90, 135, and 180; n = 20 in each group), or were left untreated (controls; n = 8). Two additional groups wore a “low magnitude” (L: +2.00DS/−4.00DC) cylindrical lens orientated at either axis 90 or 180 (n = 20 and n = 18, respectively). Refractions were measured at P5 and after 7 days of treatment for all chicks (P12), whereas videokeratography and ex-vivo eyeshape analysis were performed at P12 for a subset of chicks in each group (n = 8).

**Results:**

Compared to controls, chicks in the treatment groups developed significant amounts of refractive astigmatism (controls: 0.03±0.22DC; treatment groups: 1.34±0.22DC to 5.51±0.26DC, one-way ANOVAs, p≤0.05) with axes compensatory to those imposed by the cylindrical lenses. H cylindrical lenses induced more refractive astigmatism than L lenses (H90 vs. L90: 5.51±0.26D vs. 4.10±0.16D; H180 vs. L180: 2.84±0.44D vs. 1.34±0.22D, unpaired two-sample *t*-tests, both p≤0.01); and imposing with-the-rule (H90 and L90) and against-the-rule astigmatisms (H180 and L180) resulted in, respectively, steeper and flatter corneal shape. Both corneal and internal astigmatisms were moderately to strongly correlated with refractive astigmatisms (Pearson’s r: +0.61 to +0.94, all p≤0.001). In addition, the characteristics of astigmatism were significantly correlated with multiple eyeshape parameters at the posterior segments (Pearson’s r: -0.27 to +0.45, all p≤0.05).

**Conclusions:**

Chicks showed compensatory ocular changes in response to the astigmatic magnitudes imposed in this study. The correlations of changes in refractive, corneal, and posterior eyeshape indicate the involvement of anterior and posterior ocular segments during the development of astigmatism.

## Introduction

Astigmatism is a very common refractive error but its etiology remains elusive [1–4]. Uncorrected astigmatism not only degrades the contrast of retinal image at both distance and near, the presence of significant astigmatism with specific orientation has also been associated with amblyopia [5–8] and myopia development [9–11]. The prevalence of astigmatism usually declines during childhood [8,12]. However, in American Indian, a population known to exhibit high prevalence of significant astigmatism [13–15], the prescription of spectacles correction even during early school years did not appear to improve visual functions to normal level [16]. These findings, together with asthenopia [17], tilted optic disc [18–20], and abnormal retinal electrophysiology frequently found in astigmats [21], spur the needs for understanding the etiology of astigmatism with new approach. Although several factors including genes [22,23], ethnicity [8,24–29], nutrition [30], age [31,32], and spherical refractive errors (*i*.*e*., myopia and hyperopia) [33,34] have been associated with astigmatism in humans, the effect of environmental factor is still unclear.

Visual experience plays an important role in refractive development. In response to form deprivation and spherical defocuses, a wide variety of animal models developed refractive errors [35–40,40–44]. By the way of illustration, both chicks and macaque monkeys developed ametropia primarily axial in nature, with the former animal model responsive to a broader range of spherical defocus than the latter (-30.00D to +15.00D [45,46] vs. -3.00D to +6.00D [47]). However, could the growing eye alter its ocular components to compensate for astigmatic errors? Different laboratories have investigated this question, but the results were contradictory. An initial study in chicks showed partial compensation for optically imposed astigmatism with significant effects of axis orientation, the highest magnitudes of induced astigmatism was found when imposing oblique astigmatism, and about 50% of these induced astigmatism attributed to the cornea [45,48]. However, similar results were not replicated subsequently, in chicks [49–54] or in monkeys [55,56]. On the other hand, although the presence of astigmatism produced a slight myopic or hyperopic shift in some studies [45,49,51–53,57], it did not appear to affect the compensatory response to spherical defocus [54].

The inconclusiveness of previous studies has questioned about the capability of the eye to compensate for astigmatic errors. The primary purpose of this study was to examine how the chick eye responds to imposed astigmatism with crossed-cylindrical lenses of different axis orientations and magnitudes. The secondary purpose was to determine the correlations between refractive, corneal, and eyeshape parameters in astigmatic eyeball.

## Materials and Methods

### Animal Subjects

Eggs of White Leghorn chickens (*Gallus gallus domesticus*) were hatched in the university’s central animal facilities. The chicks were reared in a temperature controlled (22°C) animal facility on a 12-hour light/12-hour dark lighting cycle (from 7:00am to 7:00pm) with food and water provided *ad libitum*. The average light illuminance was approximately 100 lux at the chick’s eye level. Care and use of the animals were in compliance with the ARVO Statement for the Use of Animals in Ophthalmic and Vision Res and the protocol was approved by the Animal Subjects Ethics Sub-committee of The Hong Kong Polytechnic University.

### Experiments

**Visual Manipulations**. At 5-day post-hatching (P5), the chicks were randomly assigned to the treatment or control group. To impose astigmatism, a crossed-cylindrical lens (PMMA, 7.6mm base curve, 10.8mm diameter, 10.8mm optical zone; Conforma, VA, USA) of specific magnitude and axis was held in front of the right eye by using a Velcro mount, and the fellow eyes were left untreated. The optical effect of crossed-cylindrical lens has been illustrated elsewhere [54,58,59]. The opposing powers at two orthogonal meridians create no spherical power and no astigmatic power at 45° away from the principal meridians. The minus-cylindrical axis was carefully oriented for individual treatment groups with the palpebral fissure as a horizontal reference line [60]. During the treatment period (P5 to P12), the lens was removed daily for cleansing; any scratched or cracked lens was replaced immediately. If the lens was found detached, the data of the chick was excluded from further analysis.

The two experiments in this study determined whether and how the orientation and magnitude of optically imposed astigmatism altered early eye growth. In experiment A, the effects of the astigmatic axis on eye growth were determined by randomly assigning the chicks to wear a high magnitude (H) crossed-cylindrical lens of power +4.00DS/-8.00DC with the minus-cylindrical axis oriented at one of four axis orientations (45, 90, 135, and 180; n = 20 in each group). These groups were referred to as H45, H90, H135 and H180, respectively. These four orientations were chosen for imposing with-the-rule (“WTR”, H90), against-the-rule (“ATR”, H180), and oblique astigmatisms (H45 and H135) which are commonly found in humans. Eight age-matched chicks received no treatment served as controls. Since we found significant effects of axis orientation on various biometric parameters in experiment A, in experiment B we tested the effects of magnitude on eye growth by adding two groups of chicks with a lower magnitude (L) crossed-cylindrical power +2.00DS/-4.00DC (L90, n = 20; L180, n = 18).

**Biometric measures**. The details of refraction method have been described elsewhere [61,62]. In brief, the refractive status was measured along the pupillary axis using a modified Hartinger refractometer (Jena Coincidence Refractometer, Model 110, Carl Zeiss Meditec, Jena, Germany) in anaesthetized chicks (isoflurane inhalation, 1.0% to 1.5% in oxygen for rapid induction and low percentage of possible complications [63]). Although isoflurane administration can lead to dopaminergic alteration in human [64] and drug-induced cycloplegia in normal chick eyes [61], no significant effect on astigmatism measurements in chick has been reported [49]. After the chick was anesthetized, the palpebral fissure was aligned horizontally, and the lower eyelid was pulled down gently by using a lid retractor without causing any distortion of the refractometer mire. Refractive errors could be varied according to the size, strength and position of the lid retractor in conjunction with eyelid tension. Furthermore, because the cornea does not behave in accordance with Gauss’s law of elastic dome [65], the coupling ratio is not equal to one (*i*.*e*., the change in steep K is not the same as the change in flat K). Thus, the effect of lid retractor could affect not only the astigmatic components, but also the spherical components. Therefore the design and application of lid retractor should be treated with caution. Nonetheless, in practice, previous studies [49,66] have shown that the presence of lid retractor produced insignificant effect on both spherical-equivalent (0.20D to 0.70D) and astigmatism measurements. For each datum, three independent measurements were taken and averaged using power vector analysis [58]. The seven refractive parameters (Spherical components: spherical-equivalent, M; most hyperopic meridian, MHM; most myopic meridian, MMM; Cylindrical components: refractive astigmatism, RA; the two vector components [58], R-J0 and R-J45, and the axis) were analysed. To avoid potential effects of diurnal variations on refractive status [67–69], all measurements were taken at approximately the same time of the day (10:00am±1hr).

After measuring the refractive changes for a large number of astigmatism imposed chicks, a subset of birds (n = 8) randomly assigned to each group was used for corneal topography and eyeshape imaging. Corneal curvatures and astigmatism were measured using a custom-made videokeratography system in alert chicks (see [70] for details). The system captured Placido-ring images (*i*.*e*., the first Purkinje image) in multiple-shot mode and analysed the central 2.80mm-diameter cornea using a custom MatLab algorithm (MatLab; The MathWorks, Natick, MA. In order to rule out the potential effect of accommodation on corneal curvature, only images acquired at relaxed accommodative status were used, these images were identified from 500–1500 Placido-ring images from each eye as demonstrating the most frequently observed mean corneal curvature [70]. The average values of the corneal curvatures along the two principal meridians were calculated, assuming a corneal refractive index of 1.369 [71–73], from three good images per eye at different time points. Further, seven corneal parameters (steepest curvature, SK; flattest curvature, FK; mean curvature (average of FK and SK), MK; corneal astigmatism, CA; the two vector components, C-J0 and C-J45; and the axis, were derived for further analyses.

**Fig 1:**
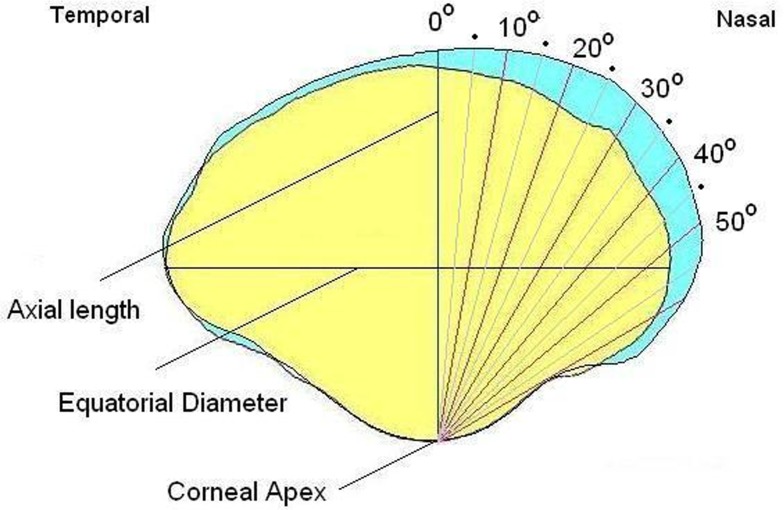
Illustration of eyeshape parameters. The eyeshape profiles of the horizontal meridian for the fellow eyes of a chick treated with +4.00DS/−8.00DCx90. The profile of the treated eye (blue area) is overlaid with that of the untreated fellow eye (yellow area). Axial length, equatorial diameter and ocular dimensions at different eccentricities (from 0° to 50° in 5° intervals), as identified by a MatLab program, are calculated with respect to the corneal apex. Identical image analysis protocol was applied to the vertical (superior and inferior regions) eyeshape profiles.

Immediately after the chicks were sacrificed by carbon dioxide asphyxiation at the end of the experiment, eyes were enucleated and eyeball profile were captured along the horizontal and vertical meridians, by an eyeshape imaging system described previously [62]. A MatLab algorithm was written to extract the following ocular dimensions by referring to the corneal apex: the axial length (AL), ocular lengths up to 50° in 5° intervals (see Fig. 1 for illustration), and equatorial diameters (vertical equatorial diameter, ED90; horizontal equatorial diameter, ED180). To study the changes in posterior eyeshape in response to cylindrical lens treatment, the inter-ocular differences in ocular dimensions between the treated/right eyes and the fellow/left eyes (*i*.*e*., treated/right eye—fellow/left eye) were calculated first from central 0° to 50° eccentricity, in 5° intervals, and summated for the horizontal (ADH, differences in area along the horizontal meridian) and vertical meridians (ADV, differences in area along the vertical meridian). We used “unit area” as a general term to represent the unit for these two quantities. The difference between ADH and ADV (ADH-ADV) was then calculated to show the meridional difference in ocular expansion of the posterior segment. In addition, the sum of ADH and ADV (ADH+ADV) was calculated to indicate the overall expansion of posterior globe.

**Internal astigmatism**. As defined previously [30,74], the internal astigmatism (IA) is the vectorial difference after subtracting the corneal astigmatism from refractive astigmatism [58,75,76].

### Data analysis

Statistical analyses were carried out using SPSS16 (SPSS, Inc, Chicago, Illinois, USA) and Oriana Version 4.01 (Kovach Computing Service). Statistical tests aimed primarily to determine the effects of crossed-cylindrical lenses on refractive, corneal and eyeshape parameters. Comparisons across groups were made by one-way ANOVAs. If the one-way ANOVA revealed significant effect, Tukey’s pairwise post hoc comparisons were used to determine which groups were significantly different. Two-sample *t*-tests were used to determine the effects of the magnitude of astigmatism between H and L groups. Watson-Williams F-tests [77,78] followed by pairwise comparisons were used to determine the treatment effects on axis orientations, the axis orientations per group were expressed as mean±angular deviation. Paired *t*-tests were used for the comparisons of parameters within eyes (*e*.*g*., horizontal vs. vertical corneal curvatures) or between treated/right and fellow/left eyes. Pearson’s correlation analyses were performed between refractive, corneal and eyeshape parameters. In all tests, significant level was set at the 95% level of confidence. Unless otherwise stated, all data were expressed in terms of inter-ocular differences (IOD) and mean±standard error (SE).

## Results

### Pre-treatment refractive status

At the onset of the two experiments, all refractive parameters (both spherical and cylindrical components) were not statistically different across the treatment and control groups (one-way ANOVA, all p≥0.40). The mean spherical equivalent (M) and refractive astigmatism (RA) in each group ranged from -0.15D to +0.27D and from -0.09D to +0.18D.

### Post-treatment effects

**Fig 2:**
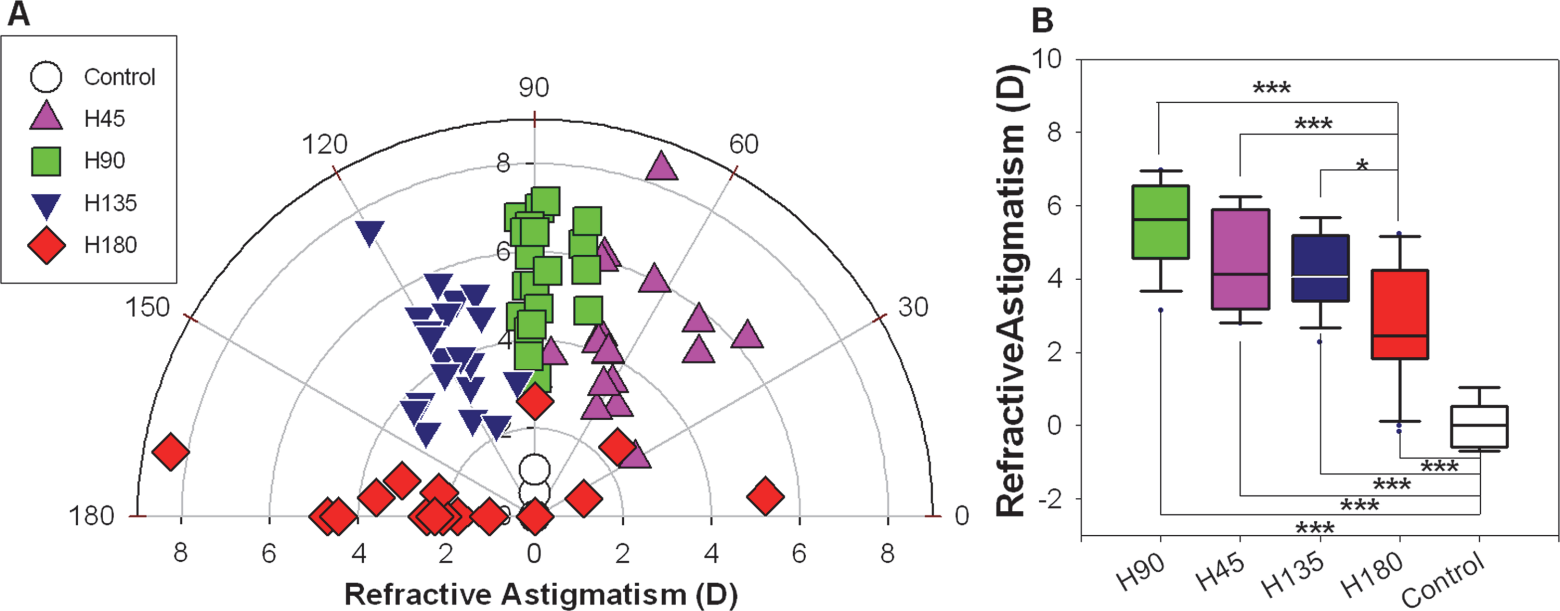
Refractive astigmatism induced by optically imposed astigmatism of four different orientations. (A) Distributions of inter-ocular differences in refractive astigmatism (treated/right eye—fellow/left eye) after one week of cylindrical lens treatment (P5-P12) for the four treatment groups (+4.00DS/-8.00DC, n = 20 in each group) with negative cylindrical axis oriented at one of the four directions (45, 90, 135, or 180), as well as the age-matched controls (n = 8). The effects of the axis of cylindrical lens are represented by different coloured symbols as shown in the legend. For example, in chicks treated with H90, the +4.00DC and -4.00DC were oriented vertically and horizontally respectively; to compensate for this astigmatic error, the eyes should develop negative cylindrical axis at 90. As shown in A, the cylindrical lenses of different axes induced compensatory astigmatism in the four treatment groups. (B) The box plots of refractive astigmatism include the values of median (line inside the box), maximum (upper whisker), minimum (lower whisker), upper (upper border of box) and lower quartiles (lower border of box) for the controls and treatment groups at P12. The levels of significant differences in the magnitudes of refractive astigmatism across the treatment groups (lines above the boxes), or between treatment and controls (lines below the boxes), are indicated by asterisk: * p≤0.05, *** p≤0.001 (Tukey’s post hoc tests).

**Refractive status − Effects of axis of astigmatism (Experiment A)**. After 1 week of treatment (P12), there was no significant difference in the spherical equivalent or most hyperopic meridian across the treatment and control groups (one-way ANOVA, both p≥0.11). However, compared to the controls, the H45 and H90 groups developed significantly more negative most myopic meridian (H45 = -3.18±0.61D; H90 = -3.19±0.30D; and Controls = -0.44±0.36D, one-way ANOVA with Tukey’s post hoc tests, both p<0.05, see Table 1). More importantly, refractive astigmatisms in the four treatment groups were all higher than those in the controls (one-way ANOVA with Tukey’s post hoc tests, all p<0.001, see Fig. 2B). As summarized in Table 1, the highest and lowest magnitudes of induced refractive astigmatism were found in the H90 group (5.51±0.26D) and the H180 group (2.84±0.44D). As shown in Table 2, the four treatment groups exhibited refractive astigmatisms of different axes (Watson-Williams F-test with pairwise comparisons, all p<0.005, see Fig. 2A); the average axes for H45, H90, H135 and H180 were, respectively, 68±7, 84±10, 119±12, and 174±44. Further analyses of the astigmatic components showed that R-J0s were significantly different between the controls and all treatment groups (one-way ANOVA with Tukey’s post hoc tests, all p<0.05), whereas R-J45s were significantly different only between the controls and the H45 and H135 groups (one-way ANOVA with Tukey’s post hoc tests, both p<0.001).

**Table 1:** Inter-ocular differences (treated/right eye–fellow/left eye) in refractive parameters (mean±SE) for the controls and treatment groups.

	Crossed-cylindrical Lens						Control
Lens Power	(H) +4.00DS/−8.00DC				(L) +2.00DS/−4.00DC		No Lens
Axis (°)	45 ^a^	90 ^a^ ^,^ ^b^	135 ^a^	180 ^a^ ^,^ ^b^	90 ^b^	180 ^b^	
n	20	20	20	20	20	18	8
M (D)	−0.94±0.64	−0.43±0.24	−0.39±0.69	+0.06±0.35	+0.29±0.17	+0.74±0.29	−0.41±0.35
MMM **(D)**	−3.18±0.61*	−3.19±0.30*	−2.53±0.66	−1.36±0.44	−1.76±0.22^#^	+0.14±0.27^##^	−0.44±0.36
MHM (D)	+1.31±0.70	+1.03±0.23	+1.75±0.74	+1.47±0.39	+2.30±0.16	+1.34±0.38	−0.42±0.34
RA (D)	4.48±0.34***	5.51±0.26***	4.29±0.27***	2.84±0.44***	4.10±0.16^#^	1.34±0.22	0.03±0.22
R-J0 (D)	−1.29±0.23**	−2.71±0.13***	−1.17±0.17*	1.22±0.25**	−2.02±0.07^##^	0.52±0.17	−0.01±0.11
R-J45 (D)	+1.47±0.21***	+0.23±0.47	−1.71±0.13***	−0.05±0.12	0.10±0.08	−0.03±0.02^#^	0.01±0.01

M = spherical-equivalent; MMM = most myopic meridian; MHM = most hyperopic meridian; RA = refractive astigmatism; R-J0 and R-J45, the two vector components of RA. In experiment A, the comparisons across the controls and treated groups were tested by one-way ANOVA followed by Tukey’s test. In experiment B, the comparisons between high and low magnitudes of imposed astigmatism were tested by two-sample *t*-tests. The levels of significant difference are indicated by asterisk: * p≤0.05, ** p≤0.01, *** p≤0.001 in experiment A, and ^#^ p≤0.05, ^##^ p≤0.01, ^###^ p≤0.001 in experiment B.

^a^ Experiment A

^b^ Experiment B

**Table 2:** Inter-ocular differences (mean±SE) in spherical equivalent (M), most myopic meridian (MMM), most hyperopic meridian (MHM), refractive (RA), corneal (CA), and internal astigmatisms (IA) for the control group and a subset of birds from the treatment groups (remark: n = 8 in each group) with both refractive and corneal measurements.

	Crossed-cylindrical Lens						Control
Lens Power	(H) +4.00DS/−8.00DC				(L) +2.00DS/−4.00DC		No lens
Axis (°)	45 ^a^	90 ^a^ ^,^ ^b^	135 ^a^	180 ^a^ ^,^ ^b^	90 ^b^	180 ^b^	Axis (°)
M (D)	-3.08±0.88	-0.78±0.40	-3.09±0.99*	0.43±0.54	0.13±0.38	0.22±0.48	-0.42±0.34
MMM **(D)**	-5.03±0.78***	-3.22±0.54*	-4.95±1.01***	-0.74±0.61	-2.12±0.42	-0.06±0.40	-0.44±0.36
MHM (D)	-1.13±1.01	1.66±0.35	-1.24±0.99	1.61±0.57	2.38±0.35	0.49±0.60	-0.42±0.35
RA (D)	3.91±0.33***	4.87±0.43***	3.71±0.31***	2.35±0.51**	4.51±0.16^#^	0.55±0.34	0.03±0.22
CA (D)	1.81±0.24**	2.27±0.22***	1.46±0.12	1.15±0.21	2.00±0.34^#^	0.74±0.14	0.60±0.18
IA (D)	2.82±0.25***	3.05±0.37***	3.18±0.35***	1.50±0.29	3.48±0.18^#^	1.01±0.18	0.72±0.16
R-J0 (D)	−1.34±0.15***	−2.36±0.20***	−0.96±0.22	1.19±0.24**	−2.21±0.07^#^	0.52±0.12	−0.01±0.11
C-J0 (D)	−0.52±0.18*	−0.91±0.11***	0.04±0.16	0.51±0.12	−0.59±0.05	0.20±0.12	0.09±0.06
I-J0 (D)	−0.81±0.13*	−1.18±0.26**	−0.80±0.20	0.67±0.17*	−1.63±0.07^##^	0.41±0.07^#^	−0.10±0.11
R-J45 (D)	1.39±0.20***	0.42±0.21	−1.52±0.22***	−0.24±0.13	0.23±0.15^#^	−0.07±0.05^#^	0.01±0.01
C-J45 (D)	0.33±0.08*	−0.08±0.17	−0.51±0.11	−0.40±0.14	−0.11±0.18	−0.20±0.10	−0.16±0.07
I-J45 (D)	1.06±0.17*	0.55±0.24	−1.10±0.29***	0.16±0.07	0.34±0.20	0.13±0.11	0.17±0.07
RA (°)	68±7	84±10	119±12*	174±44***	88±4	172±68	70±37
CA (°)	79±11**	91+6*	125±20	152±22***	96±10	117±39	109±21
IA (°)	61±10	82±21	17±15**	15±67*	82±9	92±109	1.4±49

Note that the astigmatic axes in the last three rows are calculated by circular statistics (mean±angular deviation) for the treated eyes only. In experiment A, the comparisons across the controls and treated groups were tested by one-way ANOVA followed by Tukey’s test. In experiment B, the comparisons between high and low magnitudes of imposed astigmatism were tested by two-sample *t*-tests. The levels of significant difference are indicated by asterisk: * p≤0.05, ** p≤0.01, *** p≤0.001 in experiment A, and ^#^ p≤0.05, ^##^ p≤0.01, ^###^ p≤0.001 in experiment B. Comparisons for the astigmatic axis were performed by Watson-Williams F-tests followed by pairwise comparison tests.

^a^ Experiment A

^b^ Experiment B

**Fig 3:**
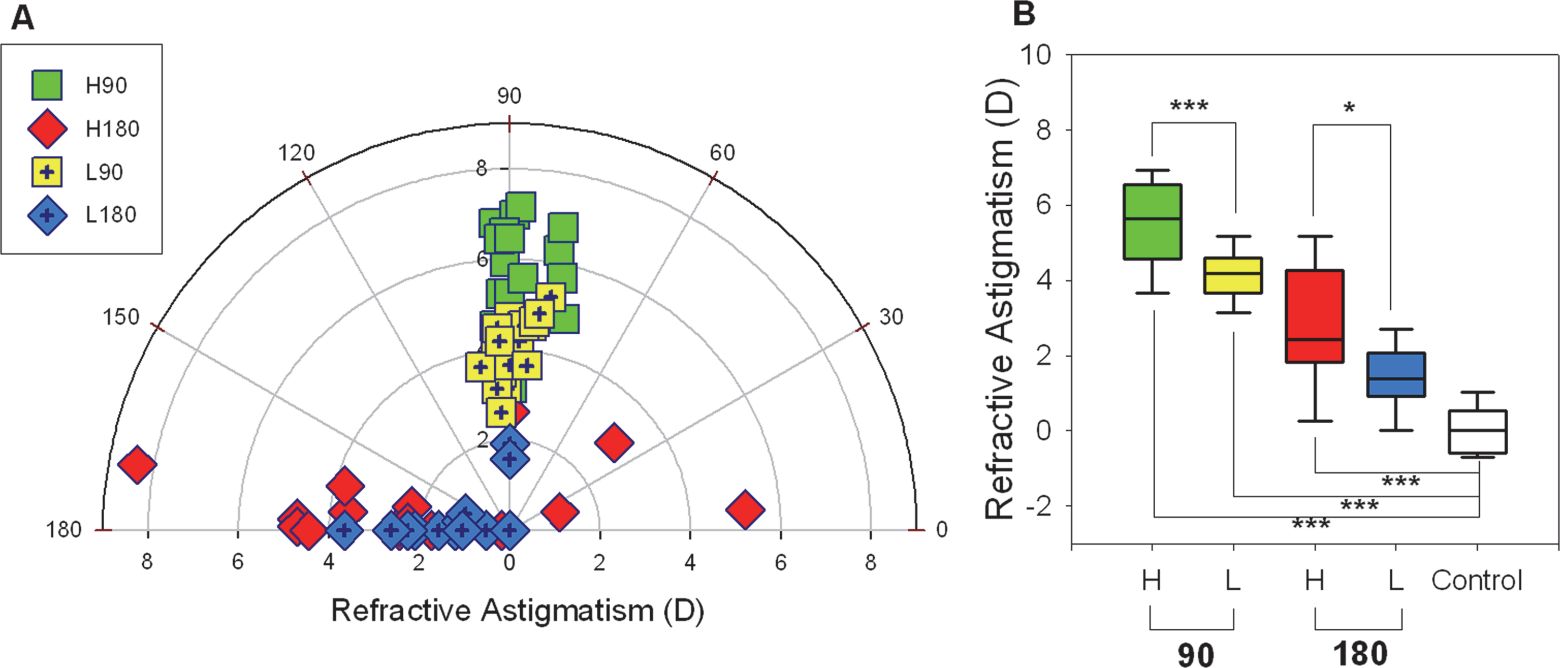
Refractive astigmatism induced by optically imposed astigmatism of two different magnitudes. (A) Distributions of inter-ocular differences in refractive astigmatism for the four treatment groups with cylindrical lenses of two magnitudes ([H]:+4.00DS/-8.00DC and [L]:+2.00DS/-4.00DC) and two axis orientations (H90, H180, and L90; n = 20 in each group; L180, n = 18). See caption for Fig. 2 and text for details. (B) The box plots of refractive astigmatism include the values of median, maximum, minimum, upper and lower quartiles for each group (see Fig. 2 for details). The levels of significant difference in the magnitudes of refractive astigmatism across the treatment groups (lines above the boxes, two-sample *t*-tests), or between treatment and control group (lines below the boxes, Tukey’s post hoc tests) are indicated by asterisk: * p≤0.05, *** p≤0.001.

**Refractive status − Effects of magnitude of astigmatism (Experiment B)**. In the two groups treated with 90° cylindrical axis, both the spherical equivalent and most myopic meridian (two-sample *t*-test, both p≤0.018), but not most hyperopic meridian (p = 0.95), were significantly different between the H90 and L90 groups. Also, both the refractive astigmatism and R-J0 (two-sample *t*-test, both p<0.001), but not R-J45 (p = 0.36), were significantly different between the H90 and L90 groups. In the other two groups treated with 180° cylindrical axis, only most myopic meridian, refractive astigmatism, and R-J0 were significantly different (two-sample *t*-tests, all p≤0.025). One-way ANOVAs (all p≤0.009) followed by Tukey’s post hoc tests (all p<0.001) showed that the H90, L90, and H180 groups developed significantly higher astigmatic components (refractive astigmatism and R-J0) than the controls. On the other hand, the axes of induced refractive astigmatism were not significantly different in both the H and L groups: H90 vs. L90 = 84±10 vs. 88±4; H180 vs. L180 = 174±41 vs. 172±58 (Watson-Williams F-test, both p≥0.39, see Table 2 and Fig. 3A).

**Corneal curvature − Effects of axis of astigmatism**. Compared to controls (0.60±0.18D), all treatment groups except the H180 group (p = 0.095) developed significantly higher corneal astigmatisms (one-way ANOVA with Tukey’s post hoc tests, all p<0.05). The highest and lowest magnitudes of corneal astigmatism among the treatment groups were found in the H90 (2.27±0.22D) and H180 groups (1.15±0.21D), respectively. The average axes of corneal astigmatism for the H45, H90, H135 and H180 groups were 79±11, 91±6, 125±20, and 152±22, respectively (see Table 2). The C-J0s of the H45 and H90 groups were significantly different from the H135, H180 groups, and the controls (One-way ANOVA with Tukey’s post hoc tests, all p≤0.037). However, C-J0s were neither significant different between the H45 and H90 groups nor among the H135, H180 and the control groups (all p≥0.116). On the other hand, the effects of obliquely oriented cylindrical axes were found between the H45 and H135 groups: the C-J45s were significantly different between the H45 and H135 groups, and between the H45 and H180 groups (one-way ANOVA with Tukey’s post hoc tests, both p≤0.001).

**Fig 4:**
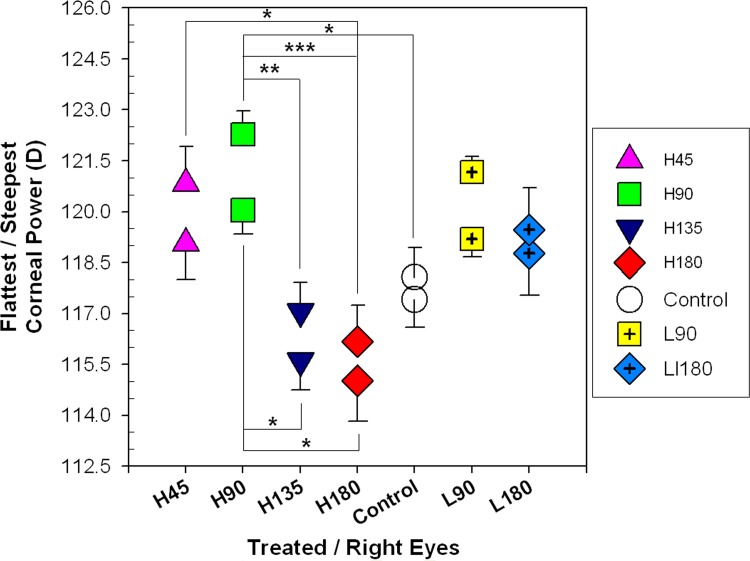
Changes in corneal curvatures in response to optically imposed astigmatism. Comparisons of the steepest (top symbols) and the flattest corneal curvatures (bottom symbols) across the controls and treatment groups at P12 (treated/right eyes data only). The levels of statistical significant difference across the treatment groups (Tukey’s post hoc tests), are indicated by asterisk: * p≤0.05, ** p≤0.01, ***p≤0.001. Although both SK and FK of L90 were not statistically different from others, they showed similar trends as those in H90.

Significant effects of cylindrical axis on corneal curvatures were found in the treated/right eyes (one-way ANOVA, all p≤0.006) but not in the fellow/left eyes (one-way ANOVA, all p≥0.241). Fig. 4 compares the steepest (top symbols) and flattest corneal curvatures (bottom symbols) of the treated/right eyes across the control and treatment groups. As shown, both SK and FK were much steeper in the H90 than other groups. Significant differences in SK were found between multiple treatment groups (H90 vs. controls, H90 vs. H135, H90 vs. H180, and H45 vs. H180; Tukey’s post hoc tests, all p≤0.05), whereas significant differences in FK were found only between H90 and the other two treatment groups (H90 vs. H135; H90 vs. H180; Tukey’s post hoc tests, both p≤0.05).

**Corneal curvature − Effects of magnitude of astigmatism**. Significant difference in C-J0 was found only between the H90 and L90 groups (two-sample *t*-test, p = 0.021). No significant differences in corneal astigmatism and C-J45 were found between the H and L groups of the same axis orientations (two-sample *t*-tests, all p≥0.055).

**Eyeshape profile − Axial length and equatorial diameter**. In general, the cylindrical-lens-wear produced an overall abnormal eyeshape. In the control group, no significant difference was found in AL (RE = 9.11mm, LE = 9.07mm), ED180 (RE = 11.99mm, LE = 11.92mm) or ED90 (RE = 12.03mm, LE = 12.08mm) between the left (LE) and right (RE) eyes (paired *t*-tests, all p≥0.191). In the treated groups, the ocular dimensions of treated eyes were significantly longer and larger than those of their untreated fellow eyes (n = 48, treated vs. fellow, AL: 9.29±0.04mm vs. 9.08±0.03mm; ED180: 12.26±0.04mm vs. 11.93±0.04mm; ED90: 12.22±0.05mm vs. 11.99±0.05mm, paired *t*-test, all p<0.001).

**Eyeshape profile − Effects of axis of astigmatism**. As shown in Table 3, the inter-ocular difference in AL of the H135 group (one-way ANOVA followed by Tukey’s post hoc test, p<0.05), as well as the inter-ocular differences in ED90 of all treatment groups (one-way ANOVA followed by Tukey’s post hoc tests, all p<0.05) were significantly larger than those of the controls. Furthermore, inter-ocular differences in ED180 of all except the H90 treatment groups were significantly larger than the controls.

**Table 3:** Inter-ocular differences in ocular dimensions (mean±SE) related to the eye shape profile for the control and treatment groups (n = 8 in each group).

	Crossed-cylindrical Lens						Control
Lens Power	(H) +4.00DS/-8.00DC				(L) +2.00DS/−4.00DC		No lens
Axis (°)	45 ^a^	90 ^a^ ^,^ ^b^	135 ^a^	180 ^a^ ^,^ ^b^	90 ^b^	180 ^b^	Axis (°)
Axial / Equatorial Dimensions							
AL (mm)	0.28±0.08	0.18±0.03	0.31±0.08*	0.21±0.05	0.16±.0.03	0.16±0.04	0.05±0.02
ED180 (mm)	0.37±.0.04**	0.25±0.05	0.34±0.06**	0.43±0.05***	0.30±0.07	0.29±0.06	0.08±0.06
ED90 (mm)	0.26±0.03**	0.30±0.06***	0.22±0.08*	0.31±0.03***	0.23±0.08	0.07±0.06	−0.06±0.08
Posterior Ocular Dimensions							
**ADH**-ADV **(unit area)**	0.10±0.70	−0.89±0.48	−0.32±0.47	2.35±0.61*	0.42±0.68	1.86±0.73	−1.02±1.01
**ADH+**ADV **(unit area)**	12.77±2.37*	7.98±1.36	12.40±3.12*	10.54±1.62	9.26±1.24	8.09±1.34	3.33±1.93
Regional Differences							
Nasal (unit area)	4.78±0.58***	3.21±0.38**	5.19±0.84***	3.93±0.45***	3.98±0.25	3.96±0.40	0.44±0.34
Temporal (unit area)	1.65±0.73	0.33±0.42	0.85±0.88	2.52±0.50	0.87±0.31	1.02±0.30	0.72±0.90
Superior **(unit area)**	3.04±0.46	1.92±0.48	3.27±0.67	1.40±0.55	2.27±0.48	1.93±0.72	1.28±0.73
Inferior (unit area)	3.30±0.86	2.52±0.41	3.09±0.83	2.69±0.52	2.51±0.47	1.18±0.47	0.89±0.68
FK (D)	−0.53±1.17	0.80±2.01	−0.85±0.63	−1.24±1.21	0.80±0.80	−0.18±0.97	−0.66±0.53
SK (D)	0.61±1.20	2.68±2.08*	−0.15±0.56	−0.65±1.05	2.03±0.87	−0.08±1.00	−0.81±0.50
MK (D)	0.04±1.17	1.74±2.04	−0.50±0.59	−0.94±1.13	1.42±0.83	−0.13±0.96	−0.74±0.51

AL = axial length; ED180 & ED90, horizontal and vertical equatorial diameters, respectively; ADH & ADV, difference in area between the two eyes up to 50° eccentricity along the horizontal and vertical meridians, respectively. In experiment A, the comparisons across the controls and treated groups were tested by one-way ANOVA followed by Tukey’s test. In experiment B, the comparisons between high and low magnitudes of imposed astigmatism were tested by two-sample *t*-tests. The levels of significant difference are indicated by asterisk: * p≤0.05, ** p≤0.01, *** p≤0.001 in experiment A, and ^#^ p≤0.05, ^##^ p≤0.01, ^###^ p≤0.001 in experiment B.

^a^ Experiment A

^b^ Experiment B

**Fig 5:**
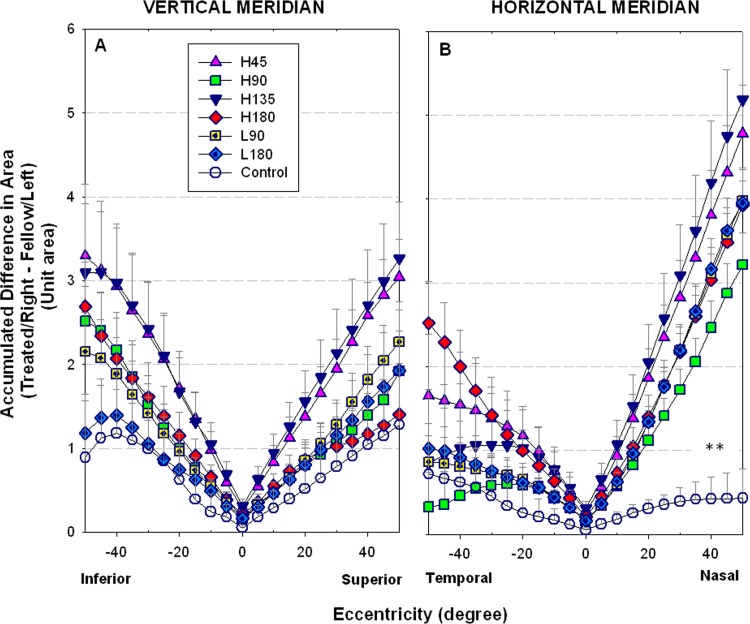
Regional changes in ocular dimensions for control and treatment groups. The regional differences in areas (treated/right eye–fellow/left eye, 5° intervals) were measured across different eccentricities along vertical (superior-inferior) (A) and horizontal (nasal-temporal) meridians (B). Note that the values at 0° showed the differences in axial length, not area. The levels of significant differences in area at eccentricity 50° between the treatment and control groups are indicated by asterisk: ** p<0.01 (Tukey’s post hoc tests).

In Fig. 5, the differences in ocular dimensions (*i*.*e*., treated/right eyes—fellow/left eyes, from 0° to 50° eccentricities in 5° intervals) towards the four peripheral regions along the vertical and horizontal meridians are compared. We found that only the nasal regions (50° eccentricities) were significant different between the controls and all treatment groups (one-way ANOVA with Tukey’s post hoc tests, all p≤0.008). To determine the effects of cylindrical lenses on posterior ocular asymmetry, we calculated the differences in area between the fellow eyes up to 50° eccentricity for the horizontal (ADH) and vertical meridians (ADV) were calculated. While the differences between the two meridians (ADH-ADV, Table 3) were analysed, we found that the H180 showed significantly larger magnitudes than the H90 and control groups (one-way ANOVA followed by Tukey’s post hoc tests, both p≤0.011). When the total changes in ocular dimensions were compared (ADH+ADV, Table 3), we found that both H45 and H135 groups had significantly larger eye sizes than the controls (one-way ANOVA with Tukey’s post hoc tests, both p≤0.041). In short, the H180 group showed meridional difference in posterior globe without an alteration in eye size; both H45 and H135 groups had larger than normal eye sizes but no differences in ocular expansion between these two meridians; whereas H90 group did not show any difference from the controls in these two parameters.

**Eyeshape profile − Effects of magnitude of astigmatism**. Significant difference in the ED90 was found only between the H180 and L180 groups (two-sample *t*-test, p = 0.002, Table 3), no significant difference in AL or ED180 was found between the H180 and L180 or between the H90 and L90 groups (two-sample *t*-tests, all p≥0.082).

**Fig 6:**
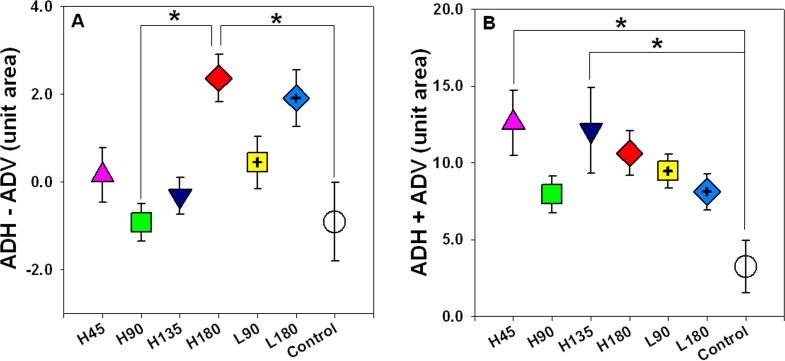
The area differences and summations. ADH and ADV indicate the area differences (treated/right eye–fellow/left eye) along vertical and horizontal meridians from 0° to 50° eccentricities. The difference (ADH−ADV) and the summation (ADH+ADV) of these parameters (mean±SE) are plotted in (A) and (B), respectively. The levels of statistical significant difference across the treatment groups (Tukey’s post hoc tests), are indicated by asterisk: * p<0.05.

When the differences in ocular dimensions (up to 50° eccentricity) at the four quadrants were analysed, the H180 group was larger than the L180 group in both the temporal and inferior regions (two-sample *t*-tests, both p<0.05, Fig. 5A and B); but no significant differences were found in all four regions between the H90 and L90 groups (two-sample *t*-tests, all p≥0.115). As shown in Fig. 6A and B, there were also no significant differences in ADH-ADV, and ADH+ADV between H180 and L180 or between H90 and L90 (two-sample *t*-tests, all p≥0.137). However, one-way ANOVA combined with Tukey’s tests revealed the (ADH-ADV) of the H180 group was larger than that of both the H90 and control groups (both p≤0.026).

**Fig 7:**
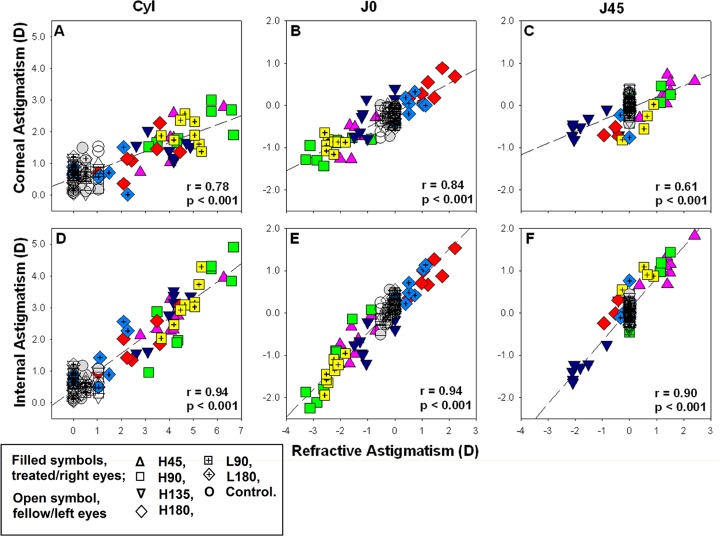
Correlations between corneal, internal and refractive astigmatisms. Data from both eyes of all treatment and control eyes (n = 112) were included in the correlation analyses for refractive and corneal astigmatic components (top panel, A-C), and for refractive and internal astigmatic components (bottom panel, D-F). Internal astigmatism is derived by calculation.

**Correlation analyses − Refractive, corneal, and internal astigmatisms**. Data from the subset of birds with both refractions and corneal topography measurements were pooled for correlation analyses (n = 112, both eyes from treated and control groups). Moderate to high correlations were found between the refractive and corneal astigmatic components (Pearson’s correlation r = 0.78, 0.84 and 0.61 for astigmatism, J0 and J45 components respectively, all p<0.001; Fig. 7A-C), as well as between the refractive and internal astigmatic components (Pearson’s r = 0.94, 0.94 and 0.90 for astigmatism, J0 and J45 components respectively, all p<0.001; Fig. 7D-F).

**Correlation analyses − Spherical components and eyeshape parameters**. Table 4 shows significant correlations found between the refractive and eyeshape parameters. The most hyperopic meridian, most myopic meridian, and spherical equivalent were significantly correlated (all p<0.001) with AL (Pearson’s r = -0.61, -0.47 and -0.57, respectively) and ADH+ADV (r = -0.40 to -0.52), but not with ED180, ED90, or ADH-ADV (all p≥0.22). All the three spherical components were significantly correlated (Pearson’s correlations, all p≤0.02) with the differences in area at superior (r = -0.36 to -0.43), inferior (r = -0.32 to -0.51), and nasal regions (r = -0.34 to -0.49); but only the most hyperopic meridian and spherical equivalent were significantly correlated (both p = 0.03) with those at the temporal region (r = -0.30 and -0.29).

**Table 4:** Pearson’s correlation analyses between refractive, corneal and eyeshape parameters (n = 56).

(D)	AL (mm)	EDmean (mm)	ED180 (mm)	ED90 (mm)	ADH (unit area)	ADV (unit area)	ADH-ADV (unit area)	ADH+ ADV (unit area)	S (unit area)	I (unit area)	N (unit area)	T (unit area)
MMM	−0.61***	−0.29*	-	-	−0.44***	−0.53***	-	−0.52***	−0.43***	−0.51***	−0.49***	-
MHM	−0.47***	-	-	-	−0.38***	−0.38***	-	−0.40***	−0.36**	−0.32*	−0.34**	−0.30*
M	−0.57***	-	-	-	−0.39***	−0.46***	-	−0.45***	−0.37***	−0.45***	−0.36**	−0.29*
RA	-	0.43***	0.28*	0.42***	-	0.33**	-	0.31*	0.27*	0.31*	0.45***	-
CA	-	0.31*	-	0.35**	-	-	-	-	-	-	-	-
IA	0.31*	0.33**	0.26*	0.32*	0.27*	0.36**	-	0.34**	0.35**	0.30*	0.45***	-
R-J0	-	-	-	-	-	-	0.36**	-	-	-	-	-
C-J0	-	-	-	-	-	-	0.35**	-	-	-	-	0.36**
I-J0	-	-	-	-	-	−0.30*	0.30*	-	−0.27*	−0.27*	-	-

M = spherical-equivalent; MMM = most myopic meridian; MHM = most hyperopic meridian; RA = refractive astigmatism; R-J0 and R-J45, the two vector components of RA; AL = axial length; EDmean = average of ED180 and ED90; ED180 & ED90, horizontal and vertical equatorial diameters, respectively; ADH & ADV, difference in area between the two eyes up to 50° eccentricity along the horizontal and vertical meridians, respectively; T, N, I and S = difference in area between the two eyes up to 50° eccentricity at temporal, nasal, inferior and superior regions, respectively. The levels of significant difference between treatment and control groups are indicated by asterisk: * p≤0.05, ** p≤0.01, *** p≤0.001.

**Correlation analyses − Astigmatic components and eyeshape parameters**. While refractive, corneal, and internal astigmatism were all significantly correlated with ED90 (Pearson’s r = 0.42, 0.35 and 0.32, respectively; all p≤0.02), only refractive and internal astigmatism were significantly correlated (all p<0.05) with ED180 (Pearson’s r = 0.28 and 0.26). In addition, both refractive and internal astigmatism were significantly correlated with the differences in area at the superior, inferior and nasal regions (Pearson’s r = 0.27 to 0.45, all p≤0.04), as well as ADH+ADV (Pearson’s r = 0.31 and 0.34, both p≤0.05). Interestingly, all the J0 components were significantly correlated with ADH-ADV (Pearson’s correlations: R-J0: r = 0.36; C-J0: r = 0.35; I-J0: r = 0.30; all p≤0.02). Furthermore, the internal astigmatism was correlated with AL (Pearson’s r = 0.31, p = 0.02), and I-J0 was correlated with the differences in area at the superior and inferior regions (Pearson’s correlations, both r = -0.27, both p = 0.04). On the other hand, although C-J0 was also correlated with the difference in area at the temporal side (Pearson’s correlation, r = 0.36, p = 0.01), corneal astigmatism was not correlated with any other parameters (all p≥0.549).

## Discussion

The main findings in this study were: 1) the chick eyes developed astigmatism after wearing crossed-cylindrical lens for a week; 2) the characteristics of the resultant astigmatism in the treated birds were influenced by the orientation and magnitude of imposed astigmatism; 3) the characteristics of the induced astigmatism were correlated with multiple eyeshape parameters.

**Fig 8:**
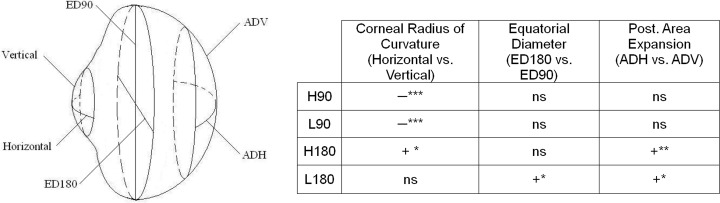
Effects of imposing WTR and ATR astigmatisms on eyeshape parameters. Comparisons of the effects of imposing WTR (H90 & L90) and ATR (H180 & L180) astigmatism on the horizontal and vertical meridians of corneal radius of curvature, equatorial diameter, and posterior ocular expansion. As illustrated in the schematic diagram, the comparisons were made for corneal curvatures of horizontal vs. vertical meridians; equatorial diameters of the horizontal (ED180) vs. vertical directions (ED90); and the difference in area up to 50° eccentricity of the horizontal (ADH) vs. vertical meridians (ADV). The table on the right summarizes the results of comparisons, the “+” and “−” signs indicate a significantly higher (horizontal>vertical) and lower (horizontal<vertical) values respectively, the “ns” represents no significant difference. The levels of significant difference, using paired *t*-test, are indicated by asterisk: * p≤0.05, **p≤0.01, *** p≤0.001.

Cylindrical lens wear also produced significant impacts on the corneal shape. The magnitudes of induced corneal astigmatism across the treatment groups varied in a similar fashion as those of the refractive astigmatism (Table 3). In the treated eyes, both the steepest and flattest corneal curvatures in the H90 group were significantly steeper than those of the H180 group (Fig. 4). Furthermore, relative to their fellow untreated eyes, imposing WTR astigmatism (H90 and L90) resulted in steeper corneal curvatures whereas imposing ATR astigmatism (H180 and L180) produced flatter corneal curvatures along both principal meridians (Fig. 4 and Table 3). As shown in Fig. 4, imposing different astigmatic axes of high magnitudes of astigmatism appeared to have a more dramatic effect on the steepest meridian: whereas significant differences in the flattest meridians were found only in three treatment groups, significant differences in the steepest meridians involved all treatment groups. It should be borne in mind that the resultant steepest meridians were oriented at different directions across the treatment groups (*e*.*g*., horizontal meridian for H90 and vertical meridian for H180), the differential magnitudes and orientations of induced astigmatism across the treatment groups suggest that the induced ocular toricity may be related to the structural anisotropy occurred regionally and/or across different meridians. Thus, the results in young chicks showed that vision-dependent processes are capable of altering corneal shape for compensation of imposed astigmatism. Several elucidations related to the ocular structures are worthy of consideration. Firstly, the corneal collagen fibrils are running in parallel to one another and oriented at orthogonal position to adjacent layers with the corneal base directly connected to the ciliary muscles [79,80]. Secondly, the anterior segment is asymmetric at the horizontal plane, with the greatest temporal distance between equator and limbus [81]. The intermediate ciliary muscle is suggested as the *depressor corneae*, with its greatest effect occurring temporally [82]. By contrast, the shortest ciliary muscle fibers, absence of intermediate ciliary muscle and poorly developed scleral venous sinus are found in the nasal quadrant. Thirdly, the overlapping patterns of the scleral ossicles (Gallus gallus: 14 ossicles, type D (1,9;6,10) pattern [83]) allow the chick cornea to alter its toricity during accommodation [84,85]. The ossicle numbers 1 (inferior) and number 9 (superior), “+” elements (on top of the others), are located nearly at the vertical meridian (i.e., the axis meridian); while the ossicle numbers 6 and 10, “-” elements (under the others), provide a buffer for the movement of the axis meridian. In combination with our previous findings of corneal accommodations [70], we speculated that the cornea could respond to imposed astigmatism, for example, the H90 group by contraction of the ciliary muscles (*i*.*e*., positive corneal accommodation), enhanced by the specific pattern of ossicles, to create an against-the-rule corneal profile (ossicles number 1&9 move forward, while number 6&10 move backward) for astigmatic compensation. Therefore, the cornea showed comparatively steeper curvatures in both principal meridians at the end of treatment, especially at the horizontal meridian due to ciliary asymmetry. As shown in Fig. 4 and 8, no significant differences in the equatorial diameters and posterior globe between the H90, L90 groups and the controls were found. Perhaps, the scleral ossicles might have sufficient flexibility to counteract the positive accommodation. The reverse is the case; the cornea compensates for the imposed ATR astigmatism, the corneal curvatures are relatively flatter as a result of negative corneal accommodation. However, the magnitude of negative corneal accommodation is smaller than that of positive corneal accommodation [70] that may be due to the limitation of the ossicles, and/or the cornea structure. Excessive negative accommodation might stretch the cornea and sclera which become flatter, along the horizontal meridian in particular. As a consequence, the magnitudes of induced astigmatism in both the H180 and L180 groups were significantly lower than the others. The corneal fibril arrangement, ciliary muscle asymmetry and the ossicle pattern provide flexibility for cornea to alter its profile, but also limitation for astigmatism compensation at the same time. Therefore full compensation was found in the L90 group, but not found in the H90 group even the magnitude of imposed astigmatism was doubled.

The refractive astigmatism found in this study was correlated moderately with the corneal astigmatism (r = 0.61 to 0.84; Fig. 7A-C) and strongly with the internal astigmatism (r = 0.90 to 0.94; Fig. 7D-F). In terms of the magnitude of refractive astigmatism, corneal astigmatism contributed to about 40% (30% to 52%) across the treatment group (Table 2). However, when the two astigmatic components (J0 and J45) were considered, the components of internal astigmatism contributed a larger proportion about 60% (51% to 70%) to the refractive astigmatism than those of corneal astigmatism in most of the treatment groups (Table 2). Our results showed that the internal astigmatism not only contributed to the induced astigmatism but also correlated with multiple eyeshape parameters including the axial length, equatorial diameters, meridional (ADH and ADV) and regional changes in ocular dimensions (see Table 4). More importantly, similar to R-J0 and C-J0, the I-J0 was also correlated with the ADH-ADV. These results suggest that the differential changes at the posterior eye segment might have altered the normal balance of internal refractive components across different meridians and contributed to ocular toricity. Although no correlations between refractive errors and thickness of retina, choroid or sclera in chicks have been reported [86], recent clinical findings suggested that internal astigmatism may correlate with retinal topography [87]. Also, several studies [86,88,89] found increase in choroidal thickness in defocus-induced hyperopic chick eyes, whereas only equatorial choroidal thickening was found in those treated with plus-cylindrical lens (plano/+10.00DS) [48]. Such regional choroidal expansion was also demonstrated in the partial form-deprived eyes followed by unrestricted vision [90]. How the retinal topography change with imposed astigmatism in chick is still open to question. It should be noted that the internal astigmatism in our study was derived from calculation; neither the curvature nor the dimension of internal ocular components was measured. Thus, it remains unclear what were the structural correlates for these internal toricities. In this respect, it has been suggested that the internal astigmatism could be due to posterior corneal astigmatism [91], the variation of refractive index in crystalline lens [92], the tilting and/or decentration of the crystalline lens with respect to the visual axis [93]. In addition, since the crystalline lens is located inside the eyeball [18,94], the nasal-temporal asymmetric ocular expansions as observed in this study (see Fig. 5) might have influenced the on-axis refractive status. The reasons for the eyes treated with imposed astigmatism of different magnitudes and axes resulted in the nasal ocular expansion remains unclear. One possibility is that the optic nerve head and pecten at the inferior-temporal quadrant of the posterior globe may restrict potential eye growth at the temporal region [95]. Another possibility is the higher ganglion cell density on the nasal retina where might be more sensitive to visual manipulation than that on the temporal side [96,97]. Future works using the latest imaging technology may help to determine the origins of the internal astigmatism.

Unlike previous studies which showed a slight myopic shift (minus-cylindrical lenses, in chicks [45,49,51–53]) and hyperopic shift (plus-cylindrical lenses, in chicks [48,49,53]; or crossed-cylindrical lenses, in chicks [54] and monkeys [55,57]), our chicks did not show a significant shift in spherical-equivalent refractive error (Table 1). Instead, in addition to the induced astigmatism, we found that the imposed astigmatism altered the eyeshape parameters and multiple eyeshape parameters were correlated with both spherical (M, MHM, and MMM) and astigmatic components (Table 4). However, the eyeshape parameters that were correlated with spherical components do not necessarily also correlated with astigmatic components and vice versa. First, whereas all spherical components (*i*.*e*., M, MHM, and MMM) were negatively correlated with axial length, only internal astigmatism was positively correlated with axial length. Second, whereas the spherical components only correlated with the ADH+ADV, the three J0 components only correlated with ADH-ADV. Third, whereas all spherical components were correlated with ADV and ADH, only RA and I-J0 were correlated with ADV or ADH. Fourth, whereas most myopic meridian was negatively correlated with the average equatorial diameter, the refractive, corneal and internal astigmatisms were positively correlated with nearly all equatorial dimensions. Thus, the different eyeshape parametric changes associated with spherical and astigmatic components as observed in this study cautious the use of conventional measure such as ocular axial length when characterizing the impacts of changes on the posterior eye segment in the development of astigmatism.

Another interesting finding from this study is the differential effects of imposing WTR and ATR astigmatism on eyeshape parameters. As summarized in Fig. 8, imposing WTR astigmatism (H90 and L90) produced significantly steeper horizontal corneal curvature than vertical curvature; imposing ATR astigmatism (H180 and L180) produced flatter horizontal corneal curvature (H180 and L180, only H180 reached statistical significance). In contrast, imposing ATR astigmatism produced significantly greater posterior ocular expansions in the horizontal than the vertical meridian, but this effect was not observed in the groups treated with WTR astigmatism. The horizontal equatorial diameter was also significantly larger in one of the ATR-treated groups (L180) but not in any of the two WTR-treated groups. Thus, optically imposed WTR and ATR astigmatisms appear to have stronger effects on, respectively, the anterior and posterior eye shapes. However, even with these contrasting effects on the different segments of the eyeball, only the most ametropic meridian in H45 and H90 was significantly different from controls, the spherical equivalent refractive error was not significantly different between the controls and any of the treatment groups (Table 1). These results suggest that, at least within the range that we tested, the ocular parametric changes in response to astigmatic error cues may be quite specific regionally and probably independent from those observed under form deprivation and spherical defocus ([41,98]). It is possible that these differential effects of specific astigmatic cues on the individual ocular dimensions had rendered the relatively lower correlations between the refractive changes and axial length (Table 4). As postulated in previous studies, the mechanisms controlling the growths of anterior chamber and vitreous chamber, [99,100], as well as those regulating the growths of equatorial diameter and axial length, [101] could be independent from each other.

After one week of cylindrical lens treatment, virtually all treated eyes developed astigmatism and the amount of induced astigmatism varied dependent on the axis orientation and magnitude of astigmatism imposed by the lenses (Fig. 2 and 3). The highest and lowest magnitudes of induced astigmatism were found, respectively, in the treatment groups that experienced a week of WTR (H90: 5.51±0.26D) and ATR astigmatisms (L180: 1.34±0.22D). Only the L90 group developed a magnitude (4.10±0.16D) and an axis (88±4) that appeared to compensate fully for the 4.00DC imposed astigmatism (Table 1). In contrast to our findings, several earlier studies using higher magnitudes of optically imposed astigmatism (10.00DC to 16.00DC, usually plano-cylindrical lenses) did not show clear compensatory astigmatic changes in chicks (Schmid & Wildsoet, 1997; Laskowski & Howland, 1996; Phillips and Collins, 2000; Thibos, 2001). However, in the pioneer study [48] that employed similar paradigm (P0 or P2 birds worn plano/+10.00DC or plano/-9.00DC for 7 days) as ours, partial compensations in refractive astigmatism were found when the plano/+10.00DC lenses were oriented at 135 (3.75±0.63D, the highest) or 45 axes (1.00±0.38D, the lowest). One possibility for this discrepancy across studies is that the visual signals and its effects imposed by the high-powered cylindrical lenses (*i*.*e*., 10.00DC) might have approached the operating limits of the sensory mechanism and/or the structural correlates. It is worth noting that we used relatively lower magnitudes of cylindrical lenses (8.00DC and 4.00DC) and each principal powered meridian only imposed either 4.00D or 2.00D of defocus. Even with these lower powers of cylindrical lenses, the chicks only compensated partially in most of our treatment groups. Many biometric parameters were not significantly different between the H and L groups suggest that even the H lenses might have approached the limits of the operating mechanisms. Another possibility is the starting age in different experiments. Since hatchling chicks typically exhibit significant amounts of natural astigmatism [49], wearing cylindrical lenses immediately after hatching might have confounded the visual error signals used for regulating refractive development. Furthermore, age-dependent anatomical changes were also noted in normal post-hatched chicks with respects to corneal flattening [72,73] and the orientation of collagen circumscribing the central cornea [102]. Thus, the differences in experimental methodology and paradigm may have been the possible reasons for the discrepancy reported in these studies. Regardless, the current study, which included a large number of animals and biometric measures from anterior to posterior ocular segment, demonstrates that chicks are capable of compensating for astigmatic error signals and the regulatory mechanism is sensitive to the axis orientation and magnitude of imposed astigmatism.

In conclusion, the current study extends our understanding of astigmatic eye growth in chick and provides new insights into the effects of optically imposed astigmatism on corneal and eyeshape parameters.

## Supporting Information

S1 Dataset Raw data from individual birds are presented in four excel worksheets: 1). Refraction, both spherical and cylindrical refractive data of both eyes at P5 and P12 of treatment and control groups are included; 2). All, including data of refraction, corneal topography and eyeshape parameters (axial length and equatorial diameters) of both eyes at P5 and P12 (n = 8 per group); 3 & 4). RE and LE, ocular length at different eccentricities (from -60° to +60°) along horizontal and vertical meridians of right eye and left eye, respectively.
(XLS)
